# Surgical cup placement affects the heating up of total joint hip replacements

**DOI:** 10.1038/s41598-021-95387-8

**Published:** 2021-08-04

**Authors:** Philipp Damm, Alwina Bender, Vivian Waldheim, Tobias Winkler, Georg N. Duda

**Affiliations:** 1 Berlin Institute of Health at Charité-Universitätsmedizin Berlin, Julius Wolff Institute, Berlin, Germany; 2 Center for Musculoskeletal Surgery, Charité-Universitätsmedizin Berlin, corporate member of Freie Universität Berlin, Humboldt-Universität zu Berlin, and Berlin Institute of Health, Berlin, Germany; 3 Berlin Institute of Health Center for Regenerative Therapies, Charité-Universitätsmedizin Berlin, corporate member of Freie Universität Berlin, Humboldt-Universität zu Berlin, and Berlin Institute of Health, Berlin, Germany

**Keywords:** Musculoskeletal system, Orthopaedics

## Abstract

The long-term success of highly effective total hip arthroplasty (THA) is mainly restricted by aseptic loosening, which is widely associated with friction between the head and cup liner. However, knowledge of the in vivo joint friction and resulting temperature increase is limited. Employing a novel combination of in vivo and in silico technologies, we analyzed the hypothesis that the intraoperatively defined implant orientation defines the individual joint roofing, friction and its associated temperature increase. A total of 38,000 in vivo activity trials from a special group of 10 subjects with instrumented THA implants with an identical material combination were analyzed and showed a significant link between implant orientation, joint kinematics, joint roofing and friction-induced temperature increase but surprisingly not with acting joint contact force magnitude. This combined in vivo and in silico analysis revealed that cup placement in relation to the stem is key to the in vivo joint friction and heating-up of THA. Thus, intraoperative placement, and not only articulating materials, should be the focus of further improvements, especially for young and more active patients.

## Introduction

Engineering technologies have helped to make total joint replacement surgery a success story. The bioengineering solution of total joint replacements has helped to revolutionize medicine by providing patients with osteoarthritis or fractured joints a fast and effective means of reconstruction of the affected joint and a return to mobility. However, despite its broad usage and various innovative developments over the last decades, aseptic loosening remains the major cause of revision in total joint arthroplasty^1–6^, mainly caused from wear at cups or inlays. However, little causal knowledge exists on which parameters affect the friction and temperature between the head and acetabular cup in vivo. Despite substantial improvements in the field of THA, the high number of revisions due to wear, mainly generated by the articulating surfaces, and out of it the aseptic loosening remains remarkably constant independent of improvements in innovative surfaces, materials or designs^4,5^. Apparently, increased friction remains the primary risk factor for wear, loosening and thus survival of joint replacements^1–6^. Especially, caused by the increasing number of young^7,8^ and more active patients^9–12^ who seek THA to treat OA, the long-term success of THA has become even more relevant today. Although increased friction is considered an essential risk, surprisingly little is known about the friction characteristics that actually occur in vivo^13–16^. Also it remains unknown to what degree friction leads to temperature increases and how the two are interlinked with the occur joint contact forces and further parameters in vivo. Aspects that impact patient-specific characteristics of intra-articular friction and eventual temperature increases have been under-researched thus far.

In contrast, in technical articulations, such as joint bearings, many of the defining parameters are well known. Joint friction is known to be defined by gliding velocity, lubricant properties, contact load magnitude, articulating materials and surface properties. In engineering THA articulations, in vitro testing has allowed us to unravel some details of joint friction in joint replacements. Specifically, the general role of gliding partners^17–22^, lubrication^21,23–29^ or temperature increases^30–34^ in model systems has been defined^18–22,26,27,35–39^. In vitro determined temperature increases of up to 5 °C (Al_2_O_3_/Al_2_O_3_) or 10 °C (CoCr/UHMWPE) have been reported^34^. Furthermore, temperature increases were measured in vivo between 40 and 43.1 °C (Al_2_O_3_/PE) using instrumented implants, with a mean of 41.2 °C after one hour of walking^40^. By using these data within an in silico modeling approach it was possible to estimate the role of the articulating implant materials and the role of THA fixation (cemented vs. noncemented)^41^. Moreover, others could estimate in vivo that temperatures could reach 47 °C if metal-on-metal pairing would be assumed^42^. However, such temperature increases would affect both the articulating surfaces (e.g. deformation and degradation of the plastic liner^43,44^) and the lubrication conditions^45^ and also result in increased surface roughness and abrasion^33^. Moreover, it was demonstrated by Fang et al. that an temperature increase of the synovial fluid can be followed by an accelerated corrosion of the metallic implant material^46^. Above, it can bias the surrounding tissues^43,44^ also negatively, and thus can exhibits a further risk for implant loosening. While these data show the relevance of the articulating implant materials and surfaces, they do not explain the found intraindividual variability of the in vivo determined temperature increases.

Despite all this work the interdependency of friction and temperature, as well as the key parameters that define both in vivo remain unknown. Hence, we hypothesized that individual implantation would be key to temperature increases and linked to the resulting joint roofing. To test this hypothesis, we developed a novel in vivo and in silico approach in a group of patients with instrumented hip implants during various activities (rehabilitation, daily and athletic activities)^16^ to identify key parameters that could explain their individual temperature increases. Second, in a prospective study design, we verified whether implant positioning, articulating kinematics or the dynamics of implant orientation drive in vivo friction and temperature increases.

## Methods

### Instrumented implants for in vivo load measurements

**Figure 1:**
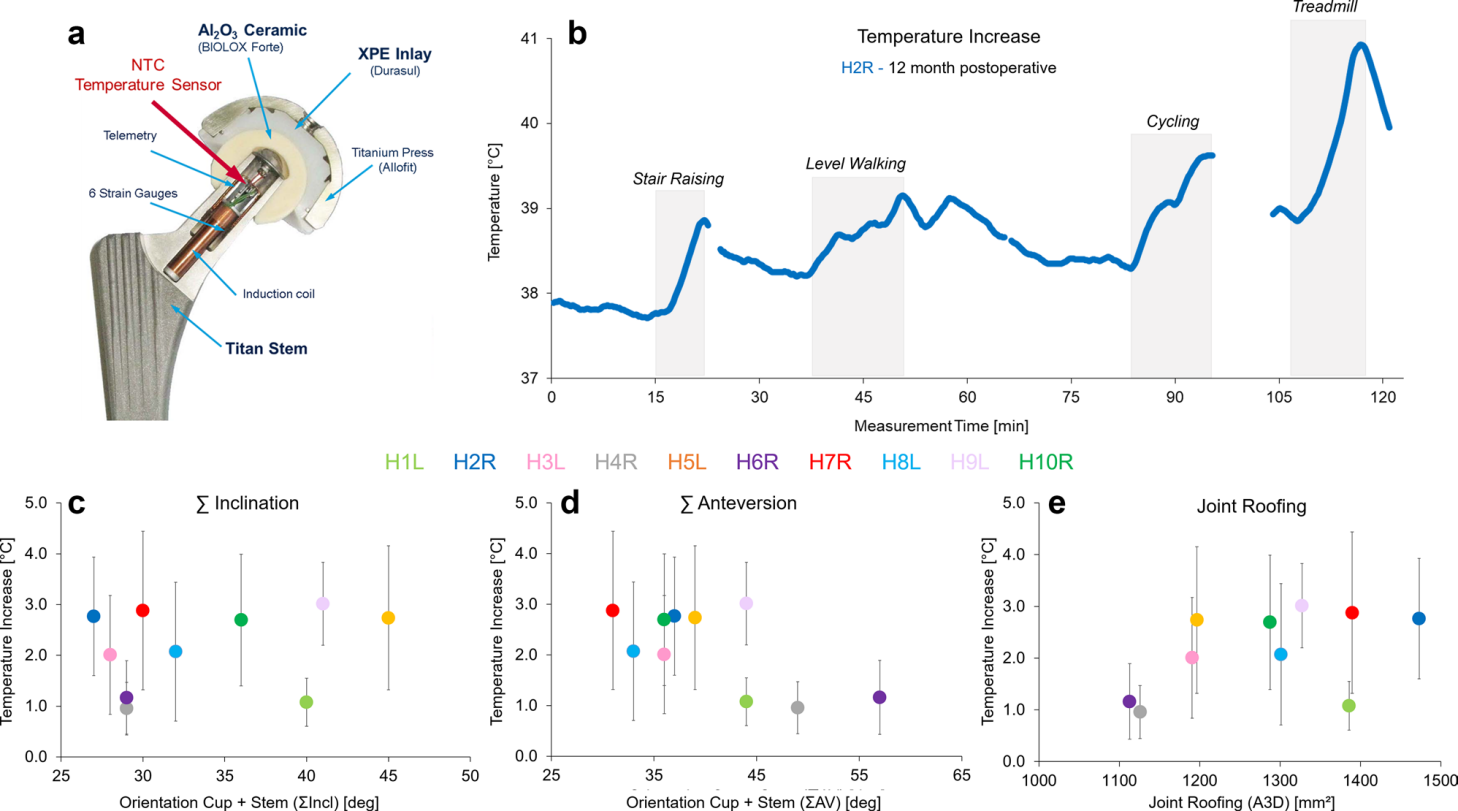
(**a**) Instrumented hip implant with an internal temperature sensor; (**b**) example of the individual pattern of the temperature increase during an in vivo load measurement session at 12 months pOP (H2R), with selected activities marked; the temperature increases (T_average_) across more than 38,000 in vivo measured trials plotted against (**c**) pelvic orientation given as sum inclination angle (∑Incl); (**d**) effective anteversion by means of the sum anteversion of femur and pelvis (∑AV) and (**e**) the size of joint roofing (A3D) measured in two-legged stance.

With a specially instrumented joint replacement implant, the joint loads and temperatures can be measured in vivo^47^. Here, we used a clinically well-established hip joint prosthesis (CTW, Merete Medical, Berlin, Germany) with a titanium stem, a 32 mm (R = 16 mm) Al_2_O_3_ ceramic head (BIOLOX forte, CeramTec GmbH, Plochingen, Germany) and a cross-linked UHMWPE (XPE) inlay (Durasul, Zimmer GmbH, Winterthur, Switzerland). Details of the instrumented implant^47^ and the external measurement system^48,49^ have previously been reported and are only briefly summarized here. The hip implants were equipped with six strain gauges in the implant neck region, which were used to measure the three force and three moment components. An additional temperature sensor (NTC) was placed inside of the implant neck, close to the center of the implant head (Fig. 1a), to allow temperature compensation of the strain gauge sensors. Moreover, this allowed us to document temperature developments at the femoral head center. The sensor had an accuracy of 0.01 °C and was integrated into the internal telemetry circuits. Even though that the temperature is measured in the head center, the occurring temperature will be deviate probably only minor in the bearing contact, because of the high thermal conductivity behavior of ceramic material^41^. The load components acting relative to the implant head are measured^50^ with an accuracy of 1–2%. The femur-based coordinate system^51^ is located in the head center of a right-sided implant but is defined relative to the bone. The positive force components act in the lateral, anterior, and superior directions. From the three force components, the resultant contact force F_res_ is calculated and reported here in Newtons (N). The joint friction, which is determined as the friction moments acting clockwise around the positive axes, is reported as the resultant friction moment M_res,_ and their values are given in Nm.

### Patient cohort

The study was approved by the local ethical committee (Charité-Universitätsmedizin Berlin, EA2/057/09), registered at the ‘German Clinical Trials Register’ (DRKS00000563) and performed in accordance with the Declaration of Helsinki. Ten subjects gave informed consent to participate in this study and continued to do so over many years postoperatively. All methods were performed in accordance with the relevant guidelines and regulations.

Based on postoperative CT data, the individual cup orientation could be identified for each patient and was reported relative to the anterior pelvic plane using anatomical landmarks^52^. The stem orientation was determined relative to the axis parallel to the posterior condyle axis^53^. Afterwards, the sum anteversion angle (∑AV) and the sum inclination angle (∑Incl), as summation of the stem and cup angles to each other during two-legged stance, were calculated to characterize THA implantation in each patient individually. Table 1 gives an overview of the range of key parameters of THA implantation in the small group of patients.

**Table 1:** Investigated subjects and individual implant orientation.

Subject	Sex	Age at implantation (years)	Bodyweight at implantation (N)	∑AV (deg)	∑Incl (deg)	Measurement day—kinematic data—months pOP
H1L	M	55	716	44	40	13
H2R	M	61	736	37	27	64
H3L	M	59	902	36	28	62
H4L	M	50	834	49	29	60
H5L	F	62	853	39	45	57
H6R	M	68	824	57	29	50
H7R	M	52	931	31	30	47
H8L	M	55	785	33	32	43
H9L	M	54	1158	44	41	12
H10R	F	53	961	36	36	12

### In vivo measurements

Starting directly after rehabilitation, each subject took part in several load measurements over a relevant period of time. While hip joint loads were recorded for all patients during typical daily activities of living^13,14,53^, the corresponding temperature increases and their interindividual variations were not analyzed and are presented here for the first time. To identify the individual variations in peak temperatures for various activities, the data recorded over the last 8 years were retrospectively analyzed across the postoperative time period monitored (up to 90 months postoperatively). The measured temperature increase per activity was averaged in each subject per postoperative measurement day and over the monitored postoperative phase.

To identify the causal relationships of the individual joint roofing and the temperature increase as well as the joint friction, three-dimensional gait data (Table 1) of all subjects were used to determine the individual joint roofing’s during the two-legged stance. These areas are considered representative joint roofing (over all activities) and depend mainly on the intraoperative specified implant orientation.

However, it must be mentioned that the available in vivo data were collected during a wide range of different activities (for a detailed activity list, see Bergmann et al.^16^ and our public in vivo load database http://www.orthoload.com), with an inter- and intraindividual randomized order and different trial lengths. Hence, the collected peak temperature values are a result of several short time measurements and do not represent the absolute maximum values or how they would occur if a specific activity would have been performed continuously over a long time period.

### Unravelling the articulating dynamics of THA and their relationship to friction-induced temperature increases

To unravel the cause for the interindividual variation in temperature increase, we reanalyzed six of the ten patients in a prospective study design (Table 2). Because walking is a repetitive activity that is easily comparable interindividually, we decided to measure the patients during walking on a treadmill (4 km/h) to determine the individual absolute peak temperature. To allow comparability, all subjects rested on a chair after entering the lab until a constant joint temperature over a minimum time of 10 min was detected. Afterwards, the subjects walked until the individual increase in joint temperature settled to the specific maximum within a walking time of up to 60 min. Once a steadiness joint temperature was detected for a minimum of 5 min (to minimize the physical strain of the subject), the treadmill was stopped, and the subjects were allowed to rest on the chair to characterize the individual cooling process.

**Table 2:** Subjects participating in the long-term measurement.

Subject BMI	Measurement day (months pOP)	Bodyweight (N)	BMI (kg/m^2^)
H2R	70	816	28.1
H5L	70	763	28.3
H6R	63	862	28.3
H7R	61	915	29.1
H8L	58	958	30.8
H10R	43	972	37.7

To determine the individual joint roofing and pathway of the contact force between the articulating surfaces, three-dimensional movement data from the gait cycles were processed and combined with the in vivo load measurements during treadmill walking. However, because these data were not collected within the long-term measurement, movement data of the same activity determined at an earlier time point (Ø44 months pOP) were used (Table 1).

### Joint roofing and contact force path between articulating surfaces

Using the individual movement data (Table 1) combined with the individual implant orientation, the joint loads measured in vivo relative to the implant head were transformed into a coordinate system relative to the cup (MATLAB R2009b, MathWorks, Natick, MA, USA). Afterwards, the resultant geometrical and load direction depended joint roofing (A3D) was determined based on the orientation of the force vector F_res_ relative to the cup (F_cup_).

1 $${\text{A3D }} = \, ({18}0^\circ \, - ({\text{arccos}}(({\underline {\text{F}}}_{{{\text{cup}}}} *{\underline {\text{x}}}_{{{\text{cup}}}} )/|{\underline {\text{F}}}_{{{\text{cup}}}} |)/{9}0^\circ ) \, * \, \pi \, *{\text{r}}^{{2}}$$

x_cup_ = normal vector of the cup opening plane with the origin in the cup center, pointing medial; r = radius implant cup.

Additionally, the individual contact pathway of the load vector F_cup_, respectively whose contact points (s_c_) in the inlay surface are calculated using formula (2).

2 $${\text{s}}_{{\text{c}}} = \, ({\underline {\text{F}}}_{{{\text{cup}}}} /|{\underline {\text{F}}}_{{{\text{cup}}}} |)*{\text{r}}$$

### Coefficient of friction

To determine the in vivo friction conditions, the time-dependent three-dimensional coefficient of friction µ_res_ was calculated based on the individual measured joint forces and friction moments according to the approach of Coulomb (formula 2):

3 $$\mu_{{{\text{res}}}} = {\text{ M}}_{{{\text{res}}}} /\left( {{\text{H }}*{\text{ F}}_{{{\text{res}}}} } \right)$$

4 $${\text{with}}\;\;{\text{H }} = {\text{ R }}* \, [{\underline {\text{F}}}_{{{\text{res}}}} /{\text{F}}_{{{\text{res}}}} {-}{\text{ cos}}({\underline {\text{F}}}_{{{\text{res}}}} /{\underline {\text{M}}}_{{{\text{res}}}} ) \, * \, ({\underline {\text{M}}}_{{{\text{res}}}} /{\text{M}}_{{{\text{res}}}} )]$$

5 $${\text{and}}\;\;{\text{cos}}({\underline {\text{F}}}_{{{\text{res}}}} /{\underline {\text{M}}}_{{{\text{res}}}} ) \, = \, \left\langle {{\underline {\text{F}}}_{{{\text{res}}}} ,\;{\underline {\text{M}}}_{{{\text{res}}}} } \right\rangle /\left( {{\text{F}}_{{{\text{res}}}} *{\text{ M}}_{{{\text{res}}}} } \right)$$

A complete derivation for the calculation was already described in detail^14^.

### Joint kinematic

Based on the three-dimensional nature of the gait movement data, we identified the gliding velocity (v_res_) at the articulation of the hip joint, including the dynamics of femoral rotation relative to the pelvis (formula 6).

6 $${\text{v}}_{{{\text{res}}}} = {\text{ d}}\left( {\text{angle of rotation}} \right)/{\text{d}}\left( {\text{t}} \right)$$

### Data evaluation and statistical analyses

To average the individual load-time patterns, an established and already described time warping method^54^ was used. Based on the averaging procedure used, extreme values of the averaged load-time patterns can slightly deviate from numerically averaged numbers.

All individual temperature–time patterns were collected over the whole measurement period and reported relative to the initial ‘start temperature’ measured when patients came into the lab and had rested until a constant joint temperature over a minimum time of 10 min was detected.

### Statistical analysis

Statistical analyses were performed using SPSS (IBM SPSS, 2013); a p-value of p < 0.05 was considered significant (one-tailed Spearman-Rho and two-way ANOVA).

## Results

First, the temperature increases were determined across all measurement trials and activities of our 10 subjects with telemetric total hip joint replacements that were available for analysis (Fig. 1a). A total of 38,000 different measurement trials were identified that could be retrospectively analyzed across a wide spectrum of activities covering rehabilitation tasks and sporting activities collected across different postoperative (pOP) measurement days (for examples see public database https://orthoload.com). The number of day-long measurements varied across subjects, with only 10 measurement day-long sessions in H1L and 25 in H2R. Additionally, the amount of the activities performed during these measurement days varied interindividually. We realized that the joint temperature changes during a measurement day depended upon the length and repetitions of the activities (example in Fig. 1b). Across all patients, activities and measurement days, the individual temperature increased on average (T_average_) between 1.0 °C (H4L) and 3.0 °C (H9L).

To evaluate whether the temperature increase could potentially be linked to acetabular positioning, the sum inclination angle (∑Incl), sum anteversion angle (∑AV) and joint roofing (A3D) in the two-legged stance were assessed in a still standing posture for all patients (Fig. 1c–e). T_average_ showed a significant correlation with A3D (r = 0.61; p = 0.031), while ∑AV only showed a mild trend (r = − 0.47, p = 0.083) and ∑Incl showed no correlation (r = 0.23, p = 0.260). In summary, the retrospective analysis indicated that temperature increases may be linked to the individual positioning of the implant.

To identify in which movements and during which articulations temperature increases can be observed and how these are linked to friction and depend upon implant positioning, we reinvited patients for a prospective measurement exercise under kinematic assessments. Only with a combined 3D movement assessment, telemetric measurement and a match of the pre-existing CT data sets to the 3D kinematic assessment could a detailed link of the 3D movement kinetics to the kinematics be performed. Thus, the temperature development over time related to 3D joint contact loading and contact area articulation in the group of instrumented patients was determined. Due to the relevance of the joint roofing (A3D) in the retrospective study, we separated subjects into three pairs, corresponding to their A3D during two legged stances (Fig. 1c): (i) small A3D: H4L and H6R; (ii) median A3D: H5L, H3L, H8L, H9L, H10R and (iii) large A3D: H1L, H2R, and H7R. Out of the initial group of ten patients, six agreed to participate (Tables 1 and 2) in the prospective evaluation during walking on a treadmill up to reaching the individual maximum steady-state temperature per patient (no changes within a minimum of 5 min).

**Figure 2:**
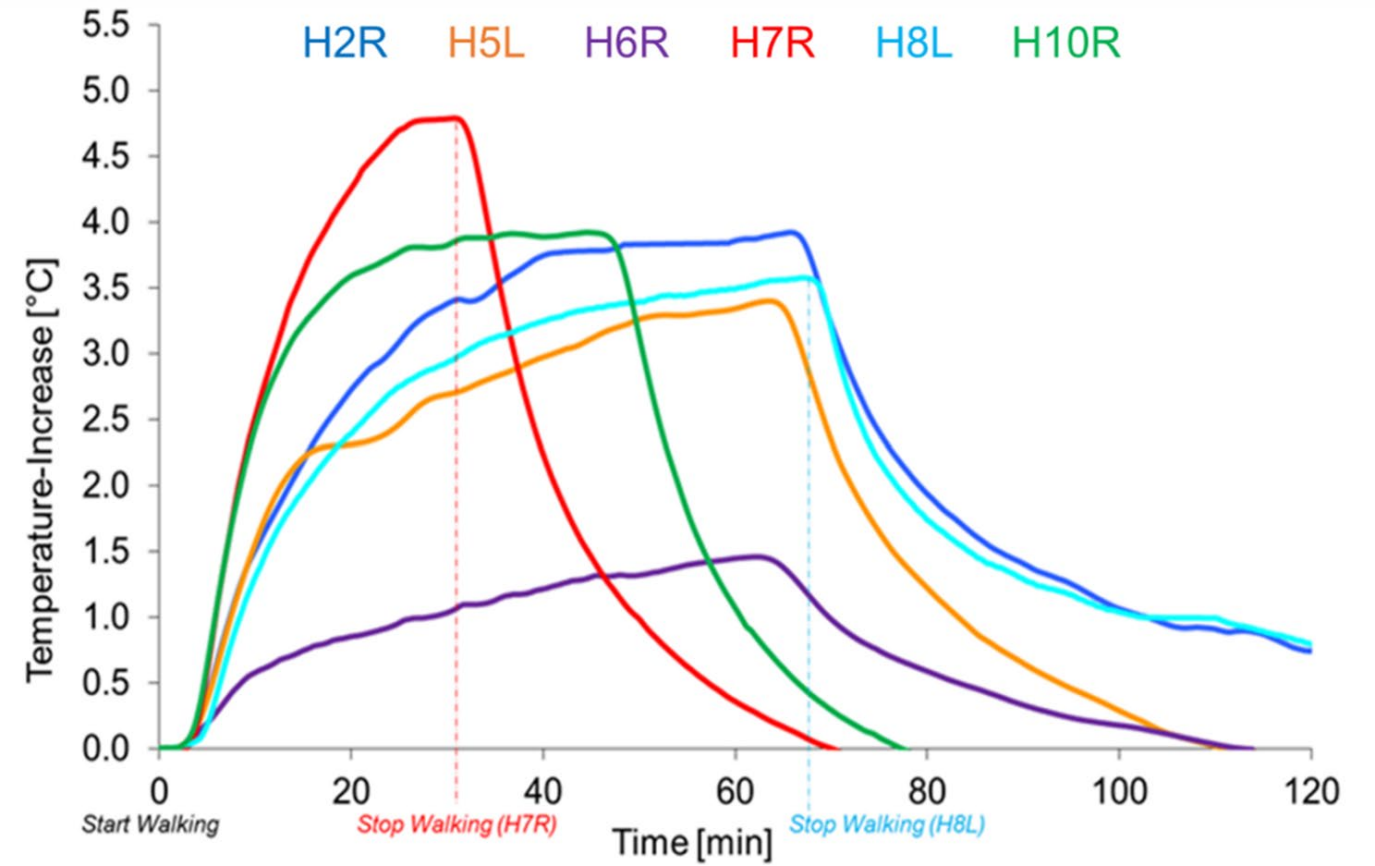
Individual in vivo measured temperature increases during treadmill walking at 4 km/h.

As in the retrospective analyses also under controlled gait at a predefined speed, substantial interindividual variations in maximum temperature were found across patients (Fig. 2). Absolute peak temperature increases (T_max_) were between 4.8 °C in H7R and 1.5 °C in H6R with very individual time curves towards a steady-state temperature increase of 3.4 °C in H5L, 3.6 °C in H8L or 3.9 °C in H2R and H10R, respectively.

To verify the findings from the retrospective observation that T_max_ was related to the individual implant positioning and the resulting size of the effective joint roofing (A3D), the CT data sets of the contact area were merged with the 3D gait kinematic assessment: T_max_ proved to be significantly influenced by the anteversion angle between the implant stem and cup ∑AV (r = − 0.81, p = 0.025) and by the contact area between the cup and head A3D (r = 0.90, p = 0.007) but not by the cup inclination angle ∑Incl (r = − 0.15, p = 0.392). In other words, the larger the sum anteversion angle ∑AV between the stem and cup is, the smaller the joint roofing A3D and the lower the in vivo measured temperature increase T_max_.

**Figure 3:**
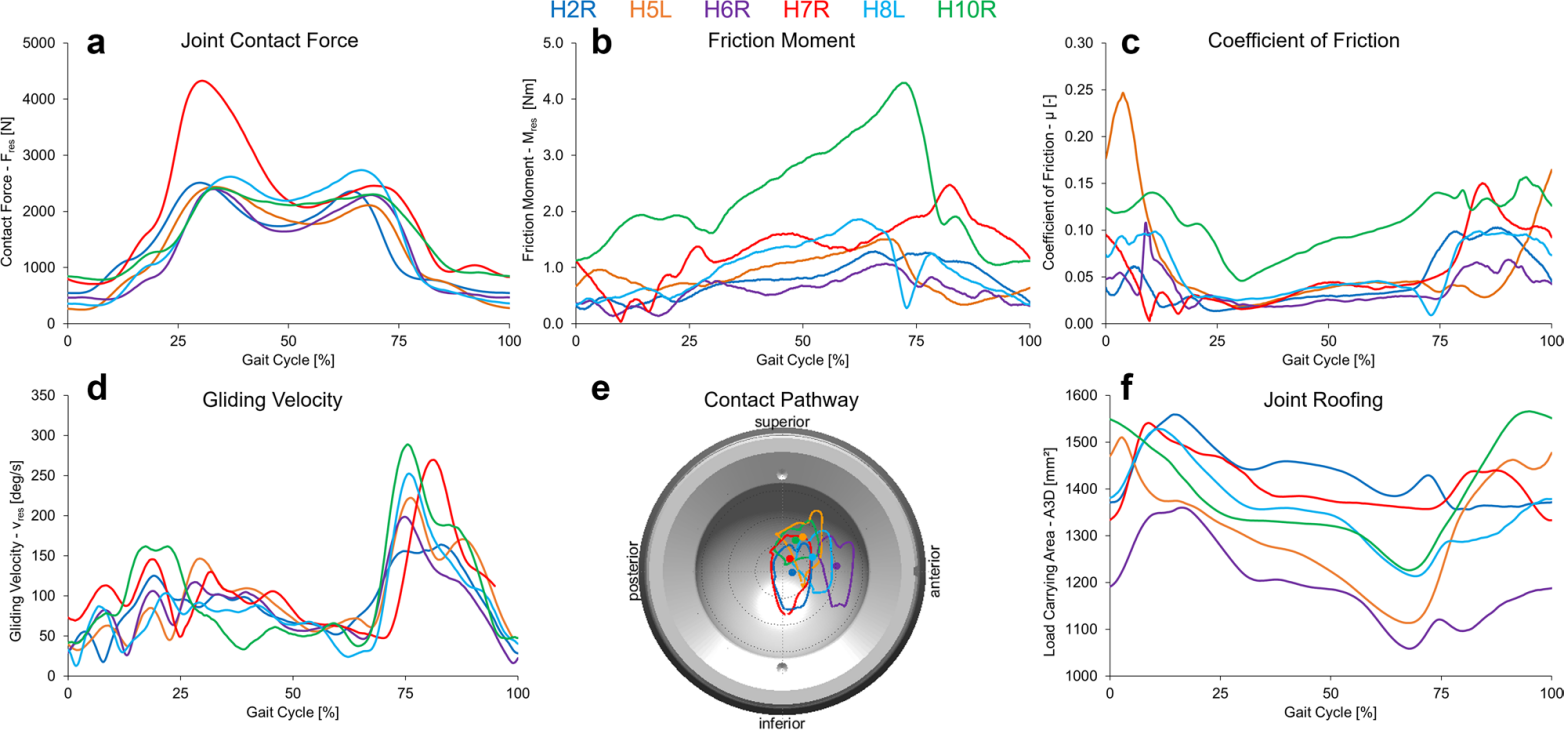
Individual pattern of the in vivo measured joint contact force F_res_ (**a**) and friction moment M_res_ (**b**) in the joint and the resultant coefficient of friction µ_res_ (**c**) the gliding velocity v_res_ (**d**), the contact pathway at the cup surface (**e**) and the resultant joint roofing A3D (**f**).

From technical articulations, we could assume that an increased joint contact force results in increased joint friction and thus also a higher temperature increase. To verify this in the group of instrumented patients, we assessed for the first time synchronous joint contact forces (F_res_), friction and temperature increase in vivo during a controlled gait task on a treadmill. In 5 subjects, contact force F_res_ (Fig. 3a) was rather comparable across patients, ranging in maximum peak values (F_max_) between 2.4 kN (H5L) and 2.7 kN (H8L). However, subject H7R showed a 73% higher maximum contact force (F_max_ = 4.3 kN). Nevertheless, F_max_ showed no significant influence on T_max_ (r = 0.67, p = 0.074) across all patients (Fig. 3a). The joint friction moment M_res_ (Fig. 3b) increased across all subjects during a gait cycle with maximum values (M_max_) between 1.0 Nm (H6R) and 1.9 Nm (H7R). Only in one subject (H10R) did the maximum joint friction moment M_max_ increase to 4.3 Nm (162% higher than average). Overall, M_max_ showed no link to F_max_ (r = 0.37; p = 0.234), a nonsignificant trend (r = 0.67, p = 0.074) to T_max_, but a significant negative correlation to ∑AV.

To allow for a step-by-step comparison, we next determined the individual coefficient of friction (µ_res_) in each subject and gait trial (Fig. 3c). Most surprisingly—and different to all in vitro observations—µ_res_ was per se not constant throughout the gait cycle but increased during the ipsilateral leg stance phase and during early swing phase. The maximum values (µ_max_) were found in the early swing phase and µ_max_ did no correlate to the maximal temperature increase T_max_ (r = − 0.04, p = 0.467) but was significantly influenced by the sum of inclination ∑Inkl (r = 0.75, p = 0.042). In one specific case, Patient H5L, µ_res_ was higher compared to all others reaching double of the average of the other patients across the whole gait cycle.

To account for the dynamic capacity to uptake the developing heat due to hip articulations into the surrounding soft tissues, the individual dynamics of temperature increase (T_Increase_) within the initial 5 min after start of the walking exercise were quantified. T_Increase_ varied between 0.4 and 0.1 °C/min and appeared to be linked to the maximal friction moment M_max_ (r = 0.93, p = 0.004) and the average movement velocity v_average_ (r = 0.93 p = 0.004). Surprisingly, a negative correlation of heat uptake was found with the anteversion angle ∑AV (r = − 0.77, p = 0.036), and no clear correlation with heat uptake was found with BMI (r = 0.60, p = 0.106). To identify the friction-induced energy uptake into the joint, the friction power (P_Friction_ = M_res _* v_gliding_) was determined. Friction power P_Friction_ showed a strong correlation with the dynamics of heat uptake by the surrounding tissues T_Increase_ (r = 0.93, p = 0.004) and with BMI (r = 0.84, p = 0.018).

Similar to heating, cooling was expected to be dominated by BMI and the respective soft tissue coverage. To determine the cooling capacities, the temperature decrease (T_Decrease_) was determined during the 5 min after completing the last walking exercise and showed values of − 0.1 °C/min to − 0.3 °C/min. T_Decrease_ appeared negatively linked to T_max_ (r = − 0.86, p = 0.014) but was also correlated to the anteversion angle ∑AV (r = 0.77, p = 0.036) and showed no clear link to BMI (r = − 0.60, p = 0.106). Thus, the cooling down T_Decrease_ appears to be more dominated by the heat conductivity of the implant components, while the friction-induced energy uptake (P_Friction_) appears to be more dominated by the implant components as well the soft tissue coverage and their heat absorption capacity. With all subjects investigated carrying the same implant material pairings with the same articulating surface properties, cooling down characteristics could be expected to be linked to the joint roofing, e.g., during sitting. However, we could not find any correlation to A3D while sitting on a chair with the cooling down phase T_Decrease_ (r = 0.53, p = 0.143).

Finally, all parameters analyzed individually that correlate to temperature development were included in interrelation analyses to identify the key factors that drive individual temperature increases in patients. A two-factorial regression analysis was performed, including all relevant key parameters that link to implant positioning (∑AV, A3D) and dynamics of articulation in vivo (contact path and position of the contact path and v_average_). In an additional analysis of variance (ANOVA), the resultant regression models were analyzed. The analyses revealed that the individual friction-induced temperature increase T_max_ was determined by a combination of only two independent key factors, an implant-related parameter and a kinematic-related parameter. Three resultant linear models were used to estimate T_max_:

(i) T_max_ (∑AV) = 4.219 − 0.096 * ∑AV + 0.032 * v_average_; r = 0.98; p = 0.009.
(ii) T_max_ (A3D) = − 13.597 + 0.01 * A3D + 0.038 * v_average_; r = 0.97; p = 0.013.
(iii) T_max_ (Position) = 0.097 − 0.249 * Position + 0.051 * v_average_; r = 0.93; p = 0.045.

In model (i), ∑AV was used as an implant-related parameter, in combination with v_average_, as a kinematic-related parameter. Alternatively, in model (ii), the joint roofing A3D was used as a reference. In model (iii), the position of the contact pathway in the liner (Position) was used as an implant-related parameter. Both the A3D and the position of the contact path in the liner are determined intraoperatively by the surgeon during implant positioning and orientation. Next to this anatomical contact definition, the gliding velocity determines temperature increases (all models i through iii). Velocity is a parameter controlled by the patient and may also be adapted by physiotherapy and patient efforts in training gait and movement.

**Figure 4:**
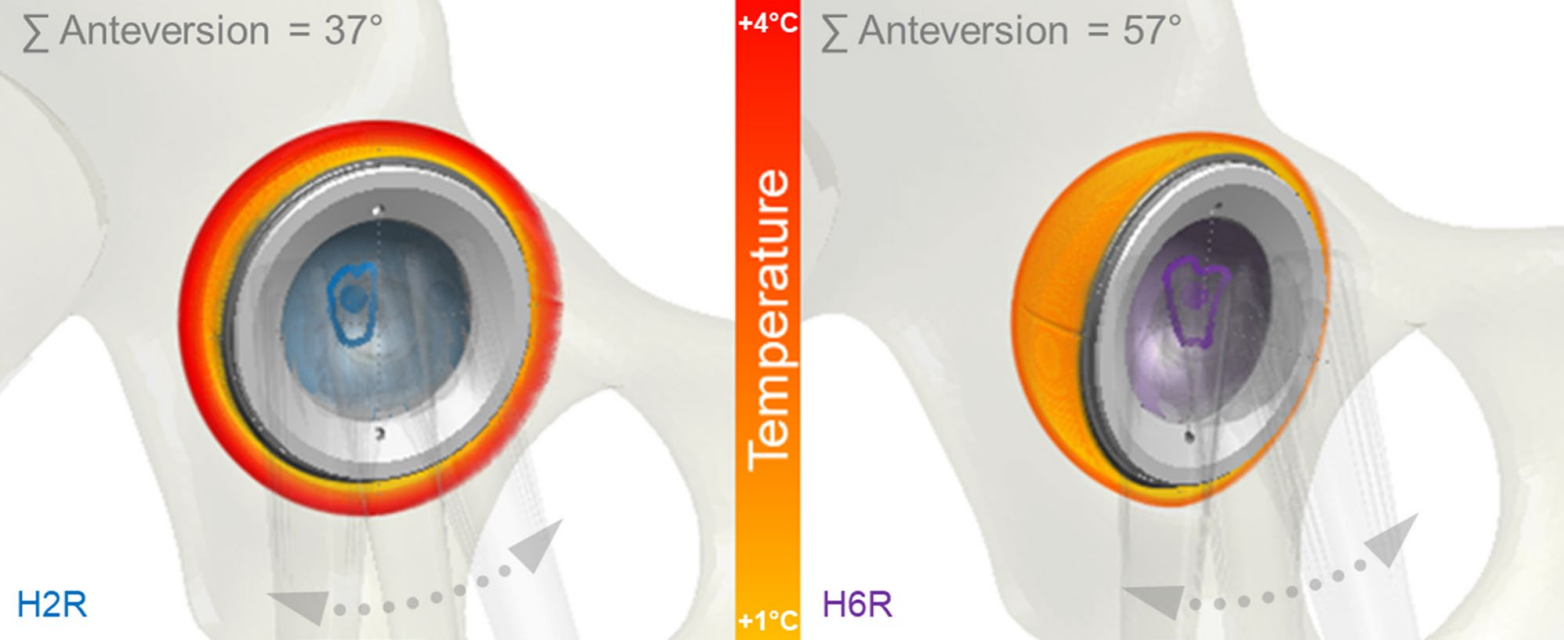
The in vivo measured temperature increase in the artificial joint replacement is determined by the individual implant orientation (∑AV), the resultant size of the joint roofing (A3D) and the position of the contact path in the liner as well as by the average gliding velocity of the joint.

Using the modeling approaches allowed us to identify that across all assumptions, the individual value of T_max_ appears to be mainly determined by a combination of the individual implant orientation ∑AV as an implantation-specific parameter (and determines A3D, the contact path and its positioning within the cup) and second by the gliding velocity selected by the patient (v_average_) and resulting from the individual kinematic chosen (Fig. 4). Only the latter parameter may be affected by training and individually controlled.

## Discussion

Joint reconstructive surgery is a highly successful medical improvement that allows millions of patients to recover after OA or femoral neck fracture and resume a normal life. During the last decade, patients who seek THA have, however, become increasingly younger, which presents this therapy with novel challenges. Over these last decades, aseptic loosening has been the dominant reason for revision surgery, and despite enormous efforts by researchers, companies and surgeons, the underlying cause that frequently leads to revision has not yet been clearly identified. With the current increase in revision cases, novel perspectives may be helpful to allow a more sophisticated view of the underlying causes of failure of total arthroplasty.

While the friction between the articulating components head and cup in a THA has long been the focus of research as a primary risk factor for survival^5,55,56^, in vivo knowledge on the friction that occurs in a real live scenario is limited. A number of in vitro and ex vivo approaches have helped to improve the articulation of THA, but despite all these analyses and respective improvements, aseptic loosening remains the primary reason for implant revision. With patients receiving artificial hip joint replacements at younger ages^7,8^ combined with higher activity levels in younger cohorts^9–12^, a better and more sophisticated understanding of the in vivo conditions that cause wear and friction and may later lead to implant loosening^57–60^ is urgently needed.

Using a novel combined in vivo and in silico approach, for the first time, the joint friction and the temperature increase during walking were analyzed within a small group of ten subjects with instrumented hip implants. The in vivo friction during standard gait is by no means constant (Fig. 3b,c); rather, it increases over the course of each gait cycle^13–15^. The data presented here show that friction (Fig. 3b) and the resulting increase in temperature (Fig. 2) are significantly determined by the implant orientation defined during the intraoperative procedure and the gliding velocity, as determined by the patient gait (Fig. 3d,f). The peak values of temperature increases depend primarily on the size of the joint roofing and the contact path taken by the head in the cup (Fig. 3e), with more central loading leading to higher temperature increases. The joint roofing and the contact path in the cup are both defined by the surgeon’s choice of the intraoperative positioning of the THA (Fig. 4).

Other aspects are also relevant for gliding conditions in vivo. Unsworth^61^ theoretically analyzed the change in fluid film thickness during walking in native and artificial hip joint replacements. He postulated that at the beginning of a gait cycle (during the swing phase), the lubricating film in the hip joint will be built up and will be decreased over the gait cycle if the joint contact force increases^61^. The minimum hereby occurs around the ipsilateral toe off. During the following swing phase, the fluid is transported back into the joint space. Hence, the determined change in the in vivo measured joint friction could be explained by a decrease in the lubricating film thickness because the synovial fluid is squeezed out from the joint space^61–63^. This squeezing of the joint space depends on the joint loading and joint kinematics and on the fluid volume and viscosity.

In conclusion, we demonstrated with this in vivo and in silico approach showing that temperature increases in THA articulations are defined by gliding velocity, the size of the joint roofing and the position of the contact pathway within the cup articulation. Both later aspects are defined by the surgeon’s choice of an implant orientation: The intraoperatively determined implant orientation (cup + stem) defines the postoperative temperature development within THA (Fig. 4). Thus, next to implant material articulation, the surgeons define the later friction conditions and resulting temperature increases in THA. This is a completely new perspective and has to date not been part of considerations of THA success (or failures). More specifically, the larger the sum anteversion angle is, the greater the contact pathway shifts towards the edge of the cup, and the shorter the path for the return transport of the synovial fluid is. The level of joint friction at the beginning of the next gait cycle depends again on the return transport of the synovial fluid into the joint space.

The in vivo and in silico approach presented here is limited due to the number of patients who were available for such measurements and carry instrumented implants. With ten patients included in the retrospective analyses, the largest possible group of these patients was made available, but this group still remained very small. For the prospective analyses, six subjects agreed to participate, which further limited the study size. However, in all patients, similar trends could be observed, which makes us confident that the data are representative as a small patient group. Furthermore, prospective assessments were performed at slightly different postoperative months. However, from the retrospective analyses, we know that the patient temperature developments were stable within the observation period. Only one tribological pairing was available for analyses (Al_2_O_3_/XPE) due to the nature of the instrumented implants and the associated patient group. However, this is the globally most frequently used material pairing and is thus representative of a large group of patients.

Today, intraoperative positioning of the joint components is based on safe zones that have been defined to ensure an optimal joint roofing for daily activities and are mainly meant to reduce the risk of luxation^64–68^. However, retrospective failure analyses have shown no difference between the revision rates of cups that were implanted inside or outside these safe zones^69,70^. Particularly, because arthroplasty patients are younger and more active today and thus the requirements for the load bearing abilities and lifetime of the implants increase, also with respect to more long-lasting activities, such safe zones should be critically re-evaluated, and biological and mechanical parameters, such as joint lubrication and joint friction, should be included in revised definitions. In vivo temperature developments can impact the surrounding bone and tissue as well as the joint replacement itself. Li et al.^71^ have investigated such effects onto the surrounding tissue. They reported that approximately 20% of osteoblasts became necrotic after being exposed to 48 °C for 10 min, while they withstood at 45 °C without damage. Yoshida et al.^72^ reported that after heating the skull of rats to 48 °C for 15 min, dead osteocyte areas were found, and the formation of new bone was delayed. Moreover, Moritz and Henriques^73^ demonstrated that all osteocytes were damaged if the bone was heated up to 50 °C for only 4 min. In conclusion, the literature suggests that an increase in bone temperature over 48 °C could certainly be critical for the implant-bone interface, but it cannot be excluded that a temperature increase already above 43 °C has no negative influences^32,33,71–75^, especially when heating is repeated and perseverative. Indeed, serious heat necrosis in the surrounding soft tissues has not been observed during the revision of hip prostheses. However, in vivo temperature-induced micronecrosis around the cup socket could be a potential risk factor for cup loosening in more active and athletic patients and should be investigated in further studies.

In addition to the effects on bone structure and bone remodeling, the observed temperature increase in hip replacement can also influence the surface quality of the tribological partners as well as the lubrication conditions. Liao et al.^33^ analyzed the temperature increase in vitro using typical hip joint simulator wear tests and investigated their effect on joint friction and wear products and on the lubricant. They observed that as a consequence of heat, the proteins of the lubricants precipitated. In addition, a decrease in the lubricating ability of the fluids was found, which was followed by an increase in joint friction and a corresponding increase in joint wear. Hence, in addition to friction loading, which stresses the surface of the tribological partners and loads as torsion torques the cup-bone interface, the friction-induced temperature increase allegorizes another risk factor for the mechanical stability of the gliding partners and lifetime of artificial cup replacement.

## Conclusion

The results of our study have shown that the in vivo measured joint friction and the resultant temperature increase in hip joint replacement were primarily influenced by the individual implant orientation and implant kinematics. These were represented by the sum anteversion angle, the size of the joint roofing, the position of the contact pathway in the artificial cup surface and the occurring gliding velocity. Interestingly, joint loads were found to only play a subordinate role compared to these parameters.

Hence, it can be concluded that the smaller the sum anteversion is, the greater the joint roofing in the artificial joint replacement, and the greater the load pathway is oriented to the center of the cup replacement, the higher the in vivo joint friction and friction-induced peak temperature in the joint replacement However, it has to be mentioned that the findings of the work are based on a small group of subjects with an instrumented hip implant. To verify the findings, it is planned to investigate the found key parameter at bigger group of subjects, using new developed instrumented hip implant which was developed specifically for temperature measurements^76^.
